# Estimation of Kidney Size From Foot Length in Newborns: A Cross-Sectional Study

**DOI:** 10.7759/cureus.23352

**Published:** 2022-03-21

**Authors:** Alok Tummuri, Mohd Saeed Siddiqui, Madhurasree Nelanuthala, Pradnya M Joshi, Jagruti Subhash Mahale, Sachin Subhash Dhule

**Affiliations:** 1 Department of Pediatrics, Mahatma Gandhi Mission (MGM) Medical College and Hospital, a Constituent Unit of MGM Institute of Health Sciences, Aurangabad, IND

**Keywords:** foot size, neonate foot length, kidney size prediction, kidney dimensions, renal size

## Abstract

Background

Kidney size determination and sonographic follow-up are important in clinical diagnosis and treatment in children. Various anthropometric measurements are correlated with gestational age and birth weight and are used to identify high-risk babies in need of early interventions. Although foot length has emerged as a simple and reliable anthropometric measurement, it is not correlated with kidney size, except in the fetal period. This study was undertaken to find a correlation between foot length and kidney dimensions and estimate kidney size by finding regression equations in newborns.

Methods

We conducted a cross-sectional study and 216 newborns were enrolled at a tertiary care hospital. Foot length was measured by digital Vernier calipers and kidney dimensions were measured by ultrasonography. The Pearson correlation coefficient and simple linear regression tests were used to determine the relationship between foot length and kidney dimensions.

Results

Foot lengths and kidney dimensions were comparable in males and females as well as on the right and left sides, except for kidney length, which was found to be longer in males. Both right and left foot lengths showed highly significant (p<0.001) but small, positive correlations with corresponding side kidney length, breadth, and area, with R-values ranging from 0.2874 to 0.3668. However, the correlation between birth weight and foot length was significant, positive, and moderate (r=0.6962 and 0.6923 for right and left foot lengths, respectively). The regression equation for estimation of kidney size from foot length was obtained but the variance explained was small (e.g. R^2^=0.1325 for right kidney length). Out of 216 babies in our study, 10 babies had a renal anomaly.

Conclusions

We found a significant but small, positive correlation between foot length and kidney dimensions. Only 13.25% of the variance in kidney length was associated with foot length. Birth weight also had a significant and positive but small correlation with kidney dimensions. However, the correlation of birth weight with foot length was moderate, and a 57.14% variance in foot length was associated with birth weight. Multivariate regression analysis with more anthropometric parameters and gestational age may help in finding a better estimation of kidney size.

## Introduction

The growth and maturity of the body and all the internal organs are characteristic of childhood. The exact evaluation of kidney size is very important for clinical diagnosis and treatment. In many renal diseases, changes occur in the size and morphology of the kidneys. It is important to follow up the kidney dimensions of patients by ultrasonography in conditions such as recurrent urinary tract infections, vesicoureteric reflux, and neurogenic bladder for the evaluation of renal growth and its abnormalities such as atrophy, and hypertrophy in children. Nomograms were developed for reliable reference for kidney size in children [1-2], to assist clinical decision-making. Anthropometric measurements such as weight, height, head circumference, and mid-upper arm circumference (MUAC) are studied to find a correlation between and estimate various parameters including internal organs within the same individual. Kidney length and volume are strongly correlated with a person’s height, weight, body mass index (BMI) [3-5], and kidney width and weight in males [6]. Kidney size is also estimated using a regression equation in Korean children for use in clinical practice [7]. Gestational age is also positively correlated with kidney dimensions along with other parameters [8-10].

It is very difficult to measure all the anthropometric parameters in a newborn, especially the one who is on a ventilator, requiring minimum handling and being nursed in the incubator. The foot length can be used as an alternative measurement and was found to be useful. There is a significant degree of correlation between the foot length of newborns and birth weight, length, and head circumference [11-13].

Foot length is a simple and most accurate anthropometric measurement to assess birth weight and gestational age in newborn babies and can be used to screen for prematurity and small-for-gestation-age babies in need of early intervention [11,14]. Foot length is also used successfully to identify high-risk babies [12]. It is also found to be a simple, proxy measure that can identify babies of low birth weight [15-16].

Although foot length is very useful, its correlation with kidney dimensions has not been studied much, except in the fetal period [17]. Therefore, this study was undertaken to find a correlation between foot length and kidney size, and if possible, to find a regression equation to estimate kidney size from foot length.

## Materials and methods

Study design

This was an observational, cross-sectional study conducted at MGM Medical College and Hospital, a tertiary care center at Aurangabad (Maharashtra, India), between October 2019 and October 2021.

Study population, sampling, and sample size

Newborn babies of either sex delivered by any mode of delivery between October 2019 and October 2021 were eligible for inclusion. Babies with limb anomalies, renal agenesis, solitary kidney, or those babies whose parents were not willing and refused to continue participating were excluded. Sampling was performed by the nonprobability, and convenience methods and newborn babies were enrolled consecutively, after obtaining consent from their parents.

A sample size of 216 was calculated with a confidence level of 95% (z=1.96) and absolute precision (d) of 0.05 with a standard deviation of 0.3 cm of foot length [18].

Data collection

Foot Length Measurement

Foot length was measured with a Zhart 0-150 mm 6 Digimatic Vernier ZC-102 digital caliper (150 mm; Zhart India, Jaipur, India). To measure the foot length, the baby’s left foot is held in the examiner’s left hand and the gauge in the right hand. The heel is placed against the platform and the head of the first metatarsal is aligned parallel to the edge of the instrument. The same procedure is repeated for the right foot length. Foot length was measured in millimeters with two decimals.

Kidney Measurements

Babies were examined within one hour of a feed so that they would be soothed and relaxed. The baby was placed in the prone position and after he/she settled, the kidneys were scanned from the posterior aspect. Kidney size was measured by a qualified radiologist on duty from the department of radiology of the MGM Hospital using a Wipro GE LOGIQ F8 ultrasonography machine (Wipro GE Healthcare Pvt. Ltd., Bengaluru, India). Ultrasound measurements of both kidneys were performed and the length was measured from the superior to the inferior perimeter to obtain the maximum length in millimeters. For the kidney, breadth superficial to the deep perimeter was taken. Kidney dimensions were measured in millimeters with one decimal.

Data analysis

All the data were compiled in an Excel spreadsheet (Microsoft Corporation, Redmond, WA) and a master chart was prepared. For data analysis, BM SPSS Statistics for Windows, Version 20.0 (IBM Corp., Armonk, NY) was used. Qualitative data were expressed in terms of frequency and percentage and quantitative data were expressed in terms of mean ± SD. Demographic statistics were summarized for age, sex, location, consanguinity, mode of delivery, and religion. Paired sample student's t-test, Pearson correlation coefficient, and simple linear regression were used for statistical analysis of the data.

Ethical considerations

Ethical permission was obtained from the institutional ethical committee (MGM-ECRHS/2019/57 dated 10th October 2019). Informed consent was obtained from the parents of the babies.

## Results

In this cross-sectional study, 216 neonates were enrolled for a correlation between foot length and kidney size. Of those, 10 babies had renal anomalies, so 206 babies were included in the analysis using SPSS software version no. 20.0. Demographic statistics were calculated for age, sex, location, consanguinity, mode of delivery, and religion. Means of left and right foot lengths and left and right kidney dimensions were compared. Sex differences in foot length and kidney sizes were also analyzed. Paired sample Student's t-test was used to find any differences in the means of the left and right foot length and kidney dimensions, such as the kidney length, breadth, and area.

The demographic features are summarized in Table 1. Out of 206 babies, males (n=100,48.54%) and females (n=106,51.46%) were comparable. The majority of babies (165, 80.1%) were between one and six days of age with a mean of 4.995 ± 5.72. The majority of babies (108; 52.43%) were from rural areas. Additionally, the mode of delivery was lower segment cesarean section (LSCS) for the majority of babies (119; 57.77%), as ours is a tertiary-care referral hospital. Out of 206 babies, 107 babies (51.94%) were born in a nonconsanguineous marriage. The majority (169; 82.04%) of babies belonged to the Hindu religion.

**Table 1:** Demographic features LSCS: lower segment cesarean section

Parameter	Categories	Frequency	Percentage (%)
Gender	Male	100	48
	Female	106	51.46
Residence	Urban	98	47.57
	Rural	108	52.43
Mode Of Delivery	Normal	87	42.23
	LSCS	119	57.77
Consanguinity	Non-Consanguineous	107	51.94
	Second Degree	01	0.49
	Third Degree	98	47.51
Religion	Hindu	169	82.04
	Islam	37	17.96

Comparisons of foot lengths and kidney dimensions in males and females are summarized in Table 2. Birth weight, right and left foot lengths, and right and left kidney breadths were comparable, and the measurements were not statistically significant. Both kidneys were longer in males than females with a mean difference of 1.84 mm for the right kidney (p=.0027), and 1.42 mm for the left kidney (p=.016).

**Table 2:** Birth weight, foot length, and kidney size: a gender comparison

Parameter	Male		Female				Total	
	Mean	SD	Mean	SD	t-value	p-value	Mean	SD
Birth weight (grams)	2524.74	569.574	2560.93	484.717	0.502	0.615	2542.67	528.324
Right foot length (mm)	74.04	5.523	74.14	4.259	0.148	0.881	74.17	4.926
Left foot length (mm)	74.05	5.413	74.46	4.162	0.623	0.533	74.26	4.827
Right kidney length (mm)	40.61	7.040	38.77	4.915	2.221	0.027*	39.02	6.136
Left kidney length (mm)	40.11	5.091	38.69	3.385	2.407	0.016*	39.19	4.379
Right kidney breadth (mm)	18.84	3.518	18.77	3.146	0.154	0.877	18.61	3.331
Left kidney breadth (mm)	19.17	3.283	18.74	2.388	1.091	0.272	18.85	2.876
Note: * p < 0.05 is significant								

Comparisons of foot lengths and kidney dimensions of the right and left sides are summarized in Table 3. The foot lengths, kidney lengths, and kidney breadths of the right and left sides were comparable, and the difference in measurements was not statistically significant.

**Table 3:** Comparison of foot length, kidney length, and kidney breadth of the right and left sides

Parameter	Right		Left			
	Mean	SD	Mean	SD	t-value	p-value
Foot length (mm)	74.17	4.926	74.26	4.826	0.1953	0.4226
Kidney length (mm)	39.02	6.136	39.19	4.379	0.4313	0.3332
Kidney breadth (mm)	18.61	3.331	18.85	2.876	0.9745	0.1651
Note: p>0.05 is not significant						

The Pearson correlation coefficient was used to find the correlation between different foot lengths, birth weight and kidney lengths, kidney breadths, and kidney areas. A simple linear regression test was used to predict kidney size and calculate the regression equation. The Pearson correlation coefficient (r) between foot lengths and kidney dimensions of the same side are provided in Table 4. Both right and left foot lengths showed a highly significant (p<0.001) but a small, positive correlation with the corresponding side kidney length, breadth, and area, with r-value ranging from 0.2874 to 0.3668. Additionally, the R^2^ value and regression equations are provided in the same table. For example, the predicted right kidney length (mm) is equal to 16.154+0.3083 x right foot length (mm) (R^2^=0.1325, SEE=4.441).

**Table 4:** Correlation between foot length and kidney dimensions

Sr.No	Correlation variables	r-value	R^2^	Regression Equation	p-value
1	Right Foot Length vs. Right Kidney Length	0.3641	0.1325	y = 16.1540+0.3083x	<0.001**
2	Right Foot Length vs. Right Kidney Breadth	0.2874	0.0826	y =7.1867+0.154x	<0.001**
3	Right Foot Length vs. Right Kidney Area	0.3451	0.1191	y = 104.65-11.276x	<0.001**
4	Left Foot Length vs. Left Kidney Length	0.330	0.1089	y =18.364+0.2805x	<0.001**
5	Left Foot Length vs. Left Kidney Breadth	0.3375	0.1139	y =5.389+0.1813x	<0.001**
6	Left Foot Length vs. Left Kidney Area	0.3668	0.1345	y = 162.07-12.204x	<0.001**
Note: **p<0.001 highly significant					

**Figure 1:**
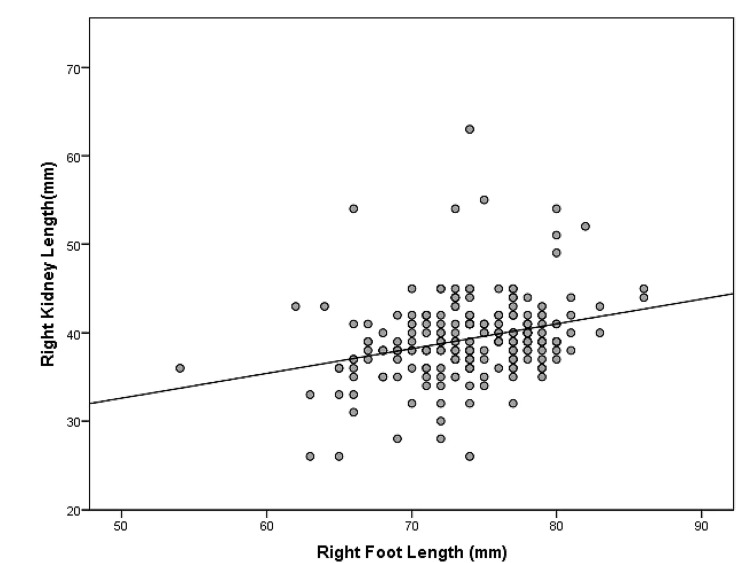
Scatter plot showing a correlation between foot length and kidney length on the right side

**Figure 2:**
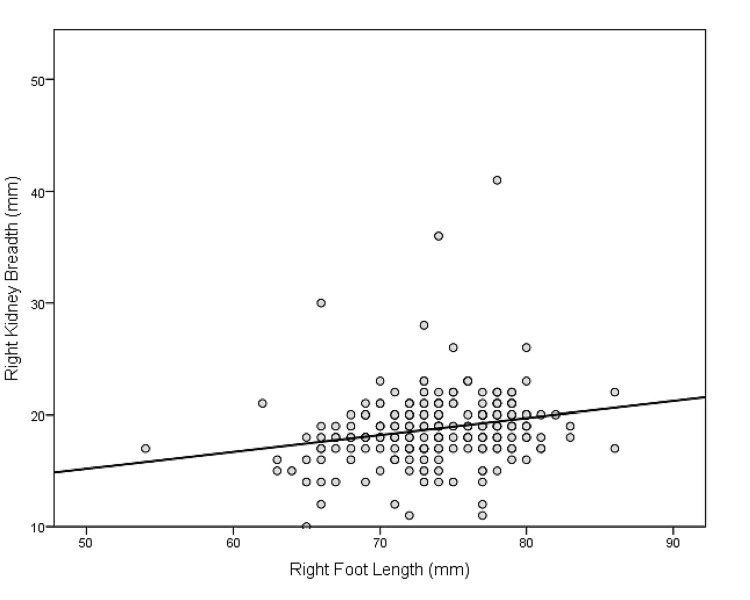
Scatter plot showing a correlation between foot length and kidney breadth on the right side

Illustrative scatter plots of the correlation between right foot length and length and breadth of the right kidney are shown in Figure 1 and Figure 2, respectively.

Correlations between birth weight and foot lengths as well as kidney dimensions are shown in Table 5. Birth weight had a highly significant (p<0.001) but small and positive correlation with kidney lengths, breadths, and areas of both sides, with R-values ranging from 0.3508 to 0. 0.4153. However, the correlation between birth weight and foot length was significant, positive, and moderate (r =0.6962 and 0.6923 for the right and left foot lengths, respectively). Additionally, the R^2^ value and regression equations are provided in the same table. For example, the predicted right foot length (mm) is equal to 55.6090+.0072 x birth weight (grams) (R^2^=0.5714, SEE=3.277). The predicted right kidney length (mm) is equal to 31.383+ 0.003 x birth weight (grams) (R^2^=0.1349, SEE=4.367).

**Table 5:** Correlation of birth weight with foot length and kidney dimensions

Sr No	Correlation variables	r-value	R^2^	Regression equation	p-value
1	Birth Weight vs. Right Foot Length	0.6962	0.5714	y = 55.609+0.0072x	<0.001**
2	Birth Weight vs. Left Foot Length	0.6923	0.5767	y = 55.762+0.0072x	<0.001**
3	Birth Weight Vs. Right Kidney Length	0.3508	0.1349	y = 31.383 +0.003x	<0.001**
4	Birth Weight Vs. Left Kidney Length	0.3634	0.1491	y =31.197+0.0031x	<0.001**
5	Birth Weight Vs. Right Kidney Breadth	0.3699	0.1534	y = 13.455+0.002x	<0.001**
6	Birth Weight Vs. Left Kidney breadth	0.3699	0.1754	y = 13.372+0.0021x	<0.001**
7	Birth Weight Vs. Right Kidney Area	0.4030	0.1796	y = 391.71+0.1328x	<0.001**
8	Birth Weight Vs. Left Kidney Area	0.4153	0.2039	y =378.27+0.1429x	<0.001**
Note: **p<0.001 is highly significant					

**Figure 3:**
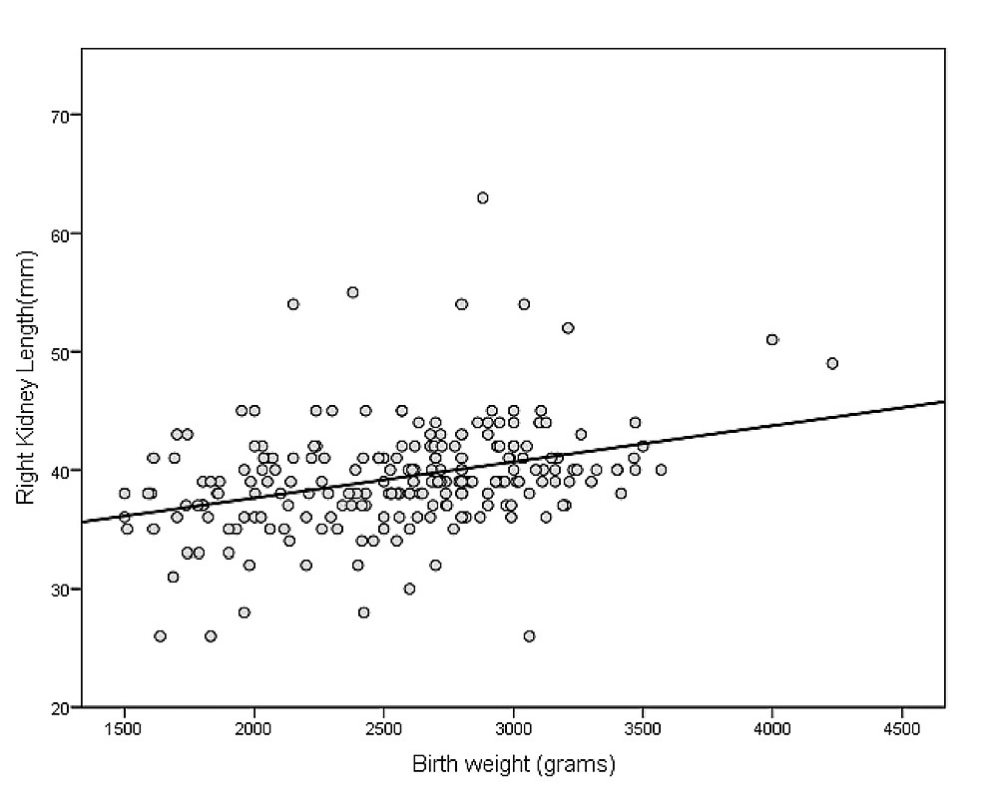
Scatter plot showing a correlation between birth weight and right kidney length

**Figure 4:**
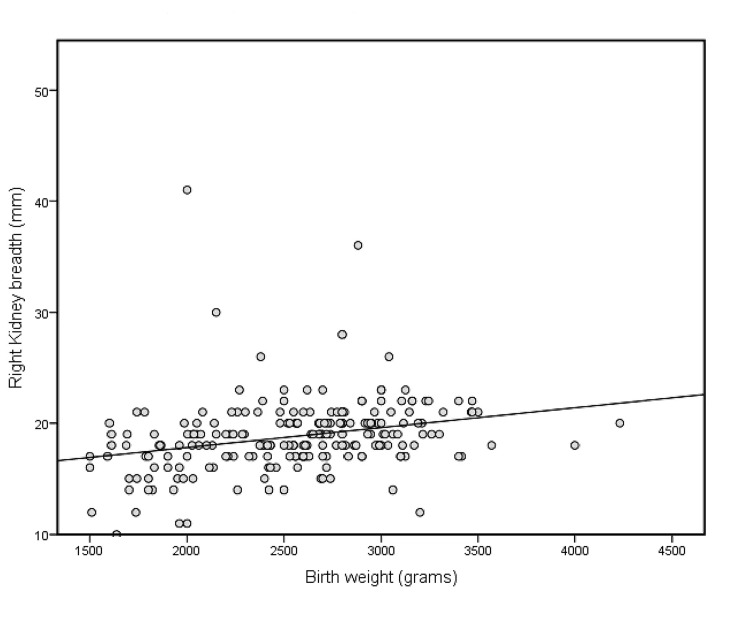
Scatter plot showing a correlation between birth weight and right kidney breadth

Illustrative scatter plots of the correlation between birth weight and the length and breadth of the right kidney are shown in Figure 3 and Figure 4, respectively.

Out of 216 in this study, 10 neonates had a renal anomaly. Five newborns had a pelviureteric junction obstruction. Two newborns had posterior urethral valves. One newborn each had a horseshoe-shaped kidney, prune belly syndrome, and vesicoureteric reflux.

## Discussion

This cross-sectional study was performed to determine the relationship between foot length and kidney size in neonates, so that the estimation of kidney size for the early detection of any abnormality may help in the early initiation of management. We found a significant but small, positive correlation between foot length and kidney dimensions. We could calculate the regression equation but the variance in kidney length explained by foot length was small (e.g., only 13.25% for the right side). Birth weight also had a significant and positive but small correlation with kidney dimensions. However, the correlation of birth weight with foot length was moderate. The regression equation explained, e.g., 57.14% of the variance in the right foot length due to variance in birth weight.

In this study, out of 216 neonates, 10 had renal anomalies so 206 newborns were included for the statistical analysis. Of 206 neonates, 106 (51.46%) were females and 100 (48.54%) were males and statistically comparable. In other studies, boys and girls were comparable in number, with a male to female ratio of 1.2:1 [19-20]. However, some studies have shown that males are more common than females [8,21-22], and a study [23] showed that the most common sex was females. These differences may be due to sampling errors and some social preferences. In the present study, 87 (42.23%) babies were born by normal delivery and 119 (57.77%) were born by LSCS. In another study, deliveries by LSCS were more common than normal deliveries [22]. As our hospital is a tertiary care center, many pregnant women are referred here for complicated deliveries. For the same reason, more (52.43%) cases were from rural areas. A greater number of mothers (82.04%) belonged to the Hindu religion, which was the majority community.

In the present study, the mean foot length was 74.17±4.926 mm and 74.26±4.826 mm on the right and left sides, respectively, and comparable (p=0.4226). The mean foot length was similar in other studies (77.2±2 mm) [24] and (77.2±5.9 mm) [8]. However in some other studies, it was different (81.9±5.5 mm) [12], (78 mm) [25], and (81±3 mm) [18] from our study. These studies are from African countries, such as Uganda and Tanzania, so ethnic differences or differences in measuring instruments may be responsible for the difference in mean foot length.

Mean kidney length and breadth in the present study were 39.02±6.136 mm, 18.61±3.331 mm on the right and 39.19±4.379 mm, 18.85±2.876 mm on the left side, and statistically comparable (p=0.332 and 0.1651, respectively). The other studies [8,17] also suggest that there is no significant difference between right and left kidney length and breadth (42.1±0.45 mm vs. 43.2±0.46 mm) [9], (41.35±1.65 mm vs. 42.17±1.63 mm ), and (38.6± 2.2 mm vs. 38.9±2.2 mm) [21]. In all studies, the left kidney size was more than the right kidney size, although the difference was not statistically significant [9,26].

Birth weight, right and left foot lengths, and right and left kidney breadths were comparable in male and female babies, and the measurements were not statistically significant (p=0.615, 0.881, 0.553, 0.887, and 0.372, respectively). Both kidneys were longer in males than females, with a mean difference of 1.84 for the right kidney (p=.0027) and 1.42 for the left kidney (p=.016), as also found in another study [1].

Both right and left foot lengths had positive and highly significant (p<0.001) but small correlations with corresponding side kidney length, breadth, and area, with R-values ranging from 0.2874 to 0.3668. On simple linear regression, we found a regression equation but the variance in kidney dimensions explained by foot length was small, ranging from 08.26% to 13.45%. For example, the predicted right kidney length (mm) is equal to 16.154+0.3083 x right foot length (mm) (R^2^=0.1325, SEE=4.441). Each mm increase in foot length was associated with a 0.30 mm increase in kidney length.

Birth weight had a positive and highly significant (p<0.001) but small correlation with kidney lengths, breadths, and areas of both sides, with R-values ranging from 0.3508 to 0. 0.4153. Similarly, in another study, kidney length and birth weight were positively correlated [21]. The predicted right kidney length (mm) is equal to 31.383 + 0.003 x birth weight (grams) (R^2^=0.1349, SEE=4.367). Each 100 g increase in birth weight was associated with a 0.3 mm increase in kidney length. However, the correlation between birth weight and foot length was highly significant (p<0.001), positive, and moderate (r =0.6962 and 0.6923 for right and left foot lengths respectively). The predicted right foot length (mm) was 55.6090+.0072 x birth weight (grams) (R^2^=0.5714, SEE=3.277). Every 100 grams increase in birth weight increased foot length by 0.72 mm. A significant correlation between birth weight and foot length was also found in other studies [13].

Out of 216 neonates in our study, 10 had a renal anomaly. Most commonly, i.e., five newborns had a pelviureteric junction obstruction leading to hydronephrosis. Two newborns had posterior urethral valves. One newborn each had a horseshoe-shaped kidney, prune belly syndrome, and vesicoureteric reflux. Other studies also demonstrated hydronephrosis as the most common (31.79%) anomaly [27].

The present study has limitations, as it was a single-center, hospital-based study and did not take the depth and volume of the kidney into consideration. As this study involved a large sample size and performing sonography by a single radiologist could not be possible, a qualified radiologist on duty performed sonography. This may introduce inter-rater measurement bias. Similarly, the mean age of measurement was 4.9 days, and we could not perform measurements at a fixed time, say 48 hours of life, due to technical issues and the willingness of parents. Additionally, the correlation of other anthropometric parameters, such as crown-rump length, MUAC, and gestational age, was not determined. Future studies need to be multicentered, and community-based and may consider using multivariate regression analysis to include other parameters in addition to foot length, such as birth weight, gestational age, MUAC, and crown-rump length, to find a better estimation of kidney size.

## Conclusions

We found a significant but small, positive correlation between foot length and kidney dimensions. Only a small variance in kidney length could be associated with foot length (e.g., 13.25% for the right side). Birth weight also had a significant and positive but small correlation with kidney dimensions. However, its correlation with foot length was moderate, and 57.14% variance in right foot length was associated with birth weight. The foot lengths and kidney dimensions of both sides and sex were not statistically significant and comparable. Only the kidney length of male babies was statistically greater than that of females. Multivariate regression analysis with more anthropometric parameters and gestational age may help in finding a better estimation of kidney size.
